# Synthesis of Photochromic Oligophenylenimines: Optical and Computational Studies

**DOI:** 10.3390/molecules20045440

**Published:** 2015-03-27

**Authors:** Armando I. Martínez Pérez, Oscar Coreño Alonso, Julián Cruz Borbolla, José M. Vásquez-Pérez, Juan Coreño Alonso, Karina Alemán Ayala, Gabriel Luna-Bárcenas, Thangarasu Pandiyan, Rosa A. Vázquez García

**Affiliations:** 1 Área de Ingeniería Mecánica Automotriz, Universidad Politécnica de Pachuca, Carretera Pachuca-Ciudad Sahagún km 20, Ex-Hacienda de Santa Bárbara, 43830 Zempoala, Hgo., Mexico; 2 Depto. Ing. Civil, Universidad de Guanajuato, Juárez 77, C.P. 36000 Leon, GTO, Mexico; 3 Área Académica de Ciencias de la Tierra y Materiales, Área Académica de Computación y Electrónica y Área Académica de Química, Universidad Autónoma del Estado de Hidalgo, Cd. Universitaria, C.P. 42184 Pachuca, Hgo., Mexico; 4 CONACyT, Avenida Insurgentes Sur 1582, Crédito Constructor, Benito Juárez, C.P. 03940 México, D.F., Mexico; 5 Centro de Investigación y de Estudios Avanzados del I. P. N., Libramiento Norponiente 2000, Fracc. Real de Juriquilla, C.P. 76230 Querétaro, Qro., Mexico; 6 Facultad de Química, UNAM, Cd. Universitaria, Circuito exterior, Coyoacán, C.P. 04510 México, D.F., Mexico

**Keywords:** mechanochemistry, oligoimines, conjugated, photochromism, DFT

## Abstract

Phenyleneimine oligomers 4,4'-(((1*E*,1'*E*)-(((1*E*,1'*E*)-(1,4-phenylenebis-(azanylylidene))bis(methanylylidene))bis(2,5-bis(octyloxy)-1,4-phenylene))bis(methanylyl-idene))-bis(azanylylidene))dianiline (**OIC1MS**) and 7,7'-(((1*E*,1'*E*)-(((1*E*,1'*E*)-((9*H*-fluorene-2,7-diyl)bis(azanylylidene))bis(methanylylidene))bis(2,5-bis(octyloxy)-1,4-phenylene))bis- (methanylylidene))bis(azanylylidene))bis(9*H*-fluoren-2-amine) (**OIC2MS**) were prepared by means of conventional and mechanochemical synthesis and characterized by FT-IR, ^1^H- and ^13^C-NMR techniques. The optical properties of the compounds were studied in solution by using UV-visible spectroscopy, and the optical effects were analyzed as a function of solvent. The results show that **OIC2MS** exhibits interesting photochromic properties. Furthermore, the structural and electronic properties of the compounds were analyzed by TD-DFT. It was found that the mechanosynthesis is an efficient method for the synthesis of both tetraimines.

## 1. Introduction

The design and synthesis of organic semiconductor devices is of interest as they can be employed as active layers in OLEDs, photovoltaic cells, transistors, smart windows, and also in photonic devices such as memory media and optical switching devices, *etc.* [1]. In these materials, conjugated polymers and oligomers are generally used due to their optical and electrical properties [2]. Photochromism occurs when isomers which can participate in reversible transformations absorb at different wavelength by alternating irradiation with UV and visible light [3].

Compounds having imine bonds act as excellent receptors and are widely used in sensors for anions in water or in biological systems, and besides conjugated imines can also be used as optoelectronic materials. Thus, it is expected that the incorporation of several imine bonds in polyaromatic compounds might result in interesting photochemical properties. Conjugated polymers like polyimines are considered for electroluminescent and photochromic-photonic devices due to their thermal stability, mechanical strength, and non-linear optical and semiconductor properties [4]. Polyimines are usually synthesized by conventional methods and also by microwave irradiation [5] and vapour deposition [6] techniques. In recent years, a novel mechanochemical synthesis (mechanosynthesis) has been considered as an efficient method for the production of some organic molecules [7] because solvents or catalysts are not required in reaction and it also avoids environmental pollution. Moreover, the reactions proceed faster than conventional syntheses. In mechanosynthesis, the reactions occur in a ball mill without external heating as the energy is being provided during the collisions between the balls or between the balls and mill walls. Thus, appropriate pressures and temperatures are reached for the reactions in the internal walls of the mill or on the balls surfaces [8].

In the present paper, two conjugated oligoimines were synthesized by means of conventional and mechanochemical synthesis, and they were characterized by spectroscopic methods. Furthermore, the optical, electronic and chemical properties were analyzed. Besides, density functional theory calculations were used to interpret the structures, optical and chemical characterization results.

## 2. Results and Discussion

### 2.1. Synthesis

**Scheme 1:**
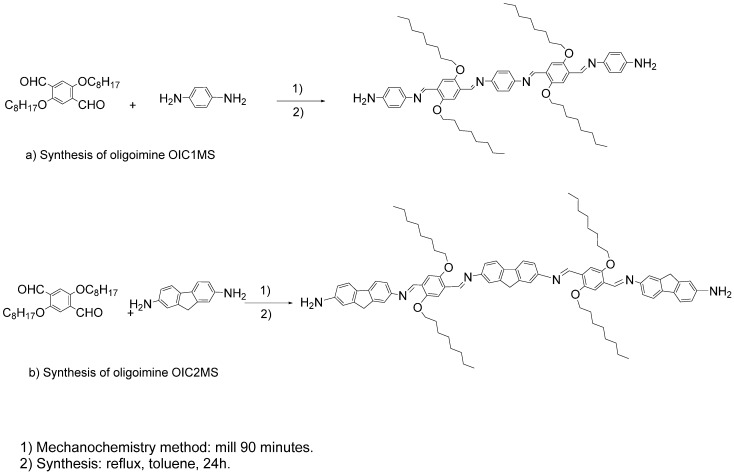
Synthesis of the conjugated oligoimines (**a**) **OIC1MS** and (**b**) **OIC2MS**.

The synthesis of the two oligoimines **OIC1MS** and **OIC2MS** was carried out by means of mechanosynthesis and the conventional method (see Scheme 1). In both methods, the formation of tetraimines with amine end-groups was observed and the yields were almost the same. However, the important difference between the two methods is that the compounds were obtained after 90 min of milling without use of inert atmosphere or solvent in the mechanosynthesis, while in the case of the conventional method, the reaction was performed under argon atmosphere for 24 h and toluene was used as solvent.

**Figure 1:**
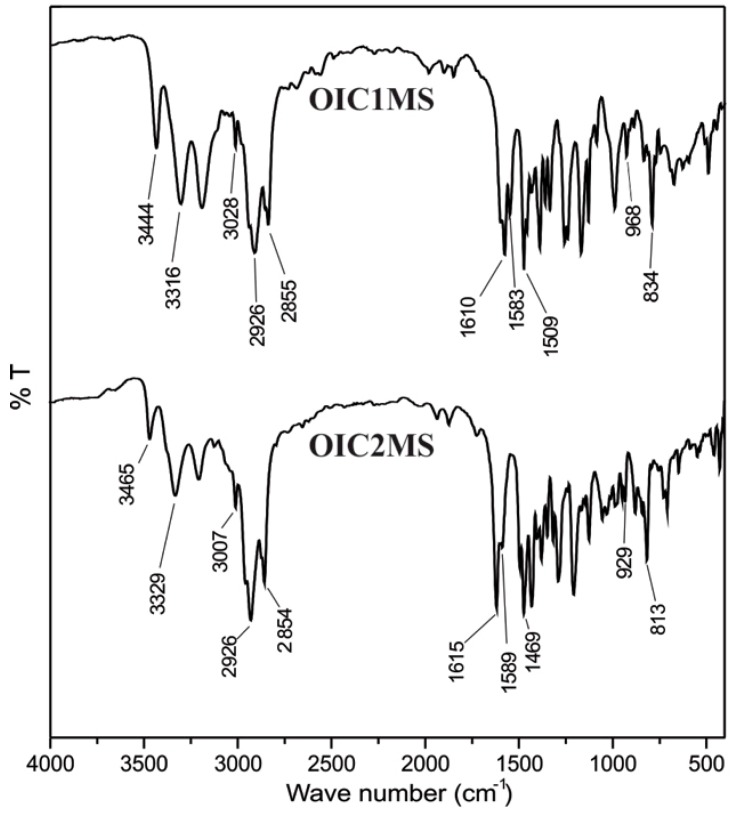
FTIR spectra of oligoimines.

**Figure 2:**
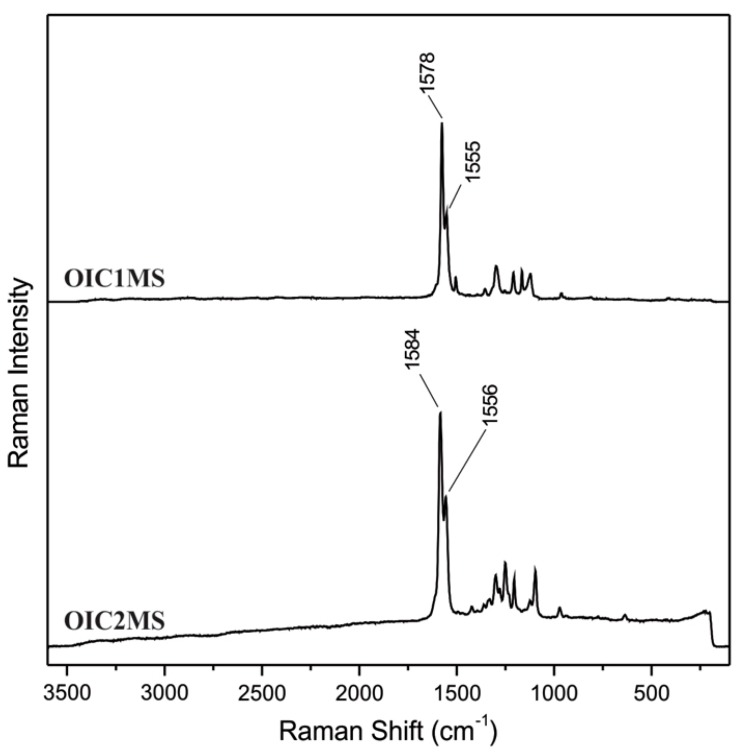
Raman spectra of oligoimines.

In the FT-IR spectrum (Figure 1) recorded for the **OIC1MS** oligomer, the bands at 3444–3316 cm^−1^ and 1583 cm^−1^ are assigned to the N-H and to the C=C bonds, respectively; the band at 1610 cm^−1^ corresponds to the imine bond (-C=N-), which can be also observed in the Raman spectra (Figure 2) at 1578 cm^−1^. Similarly, for **OIC2MS**, the bands observed at 1615 cm^−1^ in the FT-IR spectrum (Figure 1) and 1584 cm^−1^ in the Raman spectrum (Figure 2) confirm the presence of the imine bond (-C=N-) in the structure. In addition, the signals that correspond to the N-H and C=C bonds are seen at 3465, 3329 and 1589 cm^−1^, respectively.

**Figure 3:**
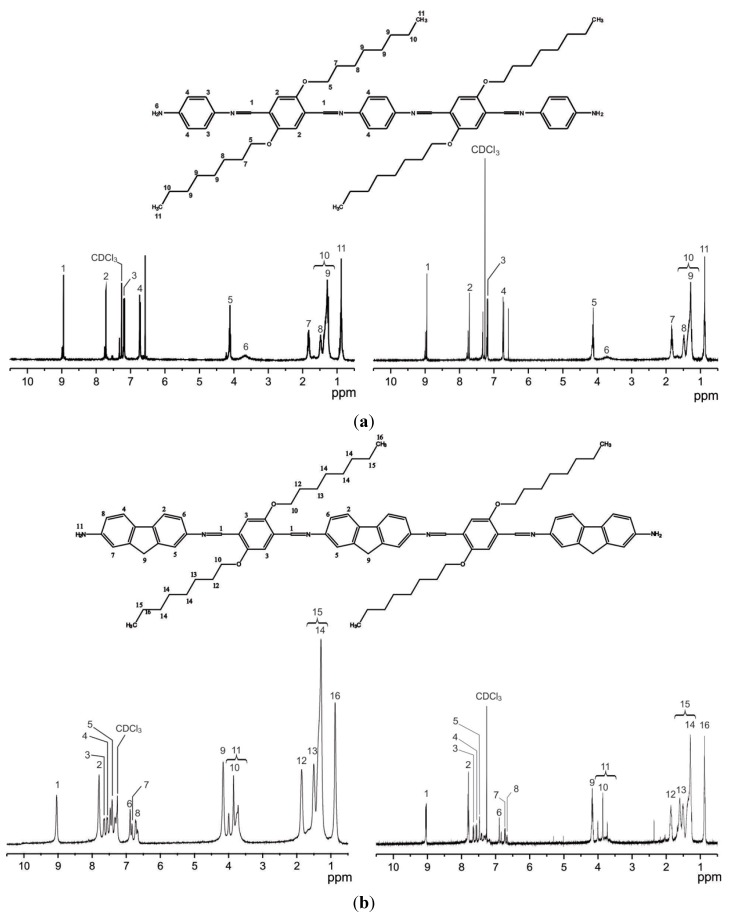
^1^H-NMR of (**a**) **OIC1MS**; and (**b**) **OIC2MS** oligomers.

In the ^1^H-NMR spectrum (Figure 3a, left) measured for **OIC1MS** the broad signal that appears at 8.9 ppm (four protons) is assigned to the imine groups (CH=N); the signal around 7.82–6.50 ppm (16 protons) corresponds to the CH group of the aromatic rings. The signal that is observed at 3.71 ppm (four protons) has been assigned to the NH_2_ terminal group in the structure. The triplet signal at 4.20 ppm (*J* = 6.7 Hz, eight protons) corresponds to the methylenes group bonded to the α-oxygen (CH_2_α-O). The signal around 1.90–1.20 ppm (48 protons) is due to the aliphatic methylene groups. Finally, the signal at 0.89 ppm with an integration of 12 protons corresponds to the terminal -CH_3_ in the molecule.

Similarly, for **OIC2MS** (Figure 3b), the broad signal at 9.03 ppm (four protons) is assigned to the imine (CH=N) bonds in the structure. The signals around 8.00–6.5 ppm (22 protons) are from the CH of aromatic rings and the broad signal at 4.16–3.54 ppm (18 protons) is assigned to the (NH_2_), (CH_2_α-O), and fluorene protons that are overlapped. The signals around 1.97–1.17 ppm (48 protons) are from the aliphatic methylene groups. The signal at 0.87 ppm (12 protons) is due to the terminal -CH_3_ groups.

Identical IR, Raman and NMR spectra were observed, irrespective of the synthesis method, showing that mechanochemical method can be used for the preparation of these compounds.

### 2.2. Optical Studies

#### 2.2.1. Absorption Spectra in Different Solvents

**Figure 4:**
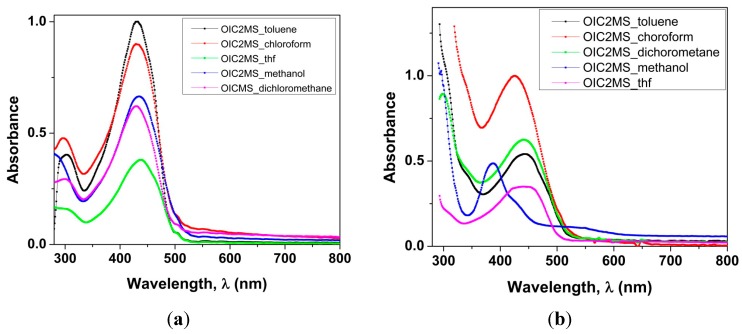
Absorption spectra: (**a**) **OIC1MS**; and (**b**) **OIC2MS** in different solvents at a concentration of 0.004 mg/ mL.

The optical properties of the compounds were analyzed in different solvents (chloroform, tetrahydrofuran, toluene, methanol and dichloromethane) by means of UV/visible spectroscopy. Results show that **OIC1MS** (Figure 4a) exhibits an absorption band at 430 nm (chloroform) which is assigned to the π-π* transition due to the conjugated bonds on the backbone [9]. It is also observed that the shift of the absorption bands is very small when the spectra are recorded in different solvents (Table 1). On the other hand, the intensity of peaks is increased in toluene as solvent. This may be due to the interaction of the toluene molecule with the conjugated oligoimines, which increases the intensity of the absorption [10]. For **OIC2MS**, (Figure 4b), a band 425 nm was observed in chloroform, which correspond to the π-π* transition on the conjugated backbone, and the intensity of bands is decreased in methanol, and also in THF. Interestingly, the intensity of peak was increased significantly in chloroform, and the wavelength 425 nm displaced better in toluene 449 nm. This observation is probably due to the fact that the conjugated system interacts effectively with toluene and increases the intensity of the band [10]. Among the two compounds, only **OIC2MS** shows a significant red-shift for band 449 nm, this can be explained by the presence of fluorene fragments in the molecule which causes more planarity promoting the intermolecular interaction.

**Table 1:** Optical properties of **OIC1MS** and **OIC2MS** in different solvents.

SOLVENT	λ_max_/nm OIC1MS	ε × 10^4^ (M^−1^ cm^−1^) OIC1MS	λ_max_/nm OIC2MS	ε × 10^4^ (M^−1^ cm^−1^) OIC2MS
Toluene	430	2.4	449	1.7
Chloroform	430	2.3	425	3.2
Methanol	434	1.7	425	1.5
Dichloromethane	430	1.6	449	2
Tetrahydrofuran	437	0.0982	440	1.1

#### 2.2.2. Photochromic Properties

**Figure 5:**
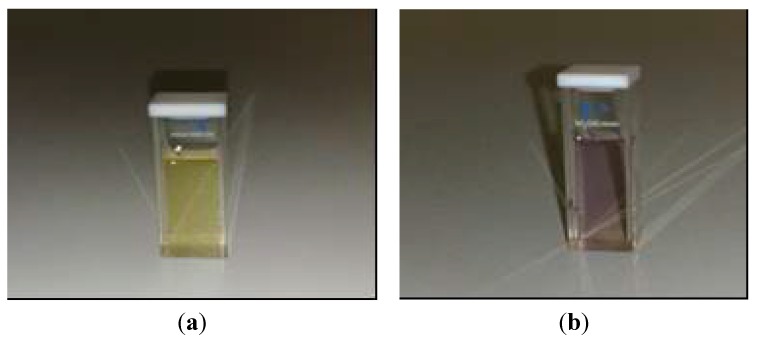
The change of color for **OIC2MS** in chloroform upon sunlight exposure. (**a**) Pale yellow solution under dark conditions and (**b**) pale violet solution after light exposure for 2 min at room temperature.

The photochromic behavior of **OIC2MS** was observed within a short time (2.0 min) after direct exposure to sunlight in chloroform at room temperature (Figure 5).

**Figure 6:**
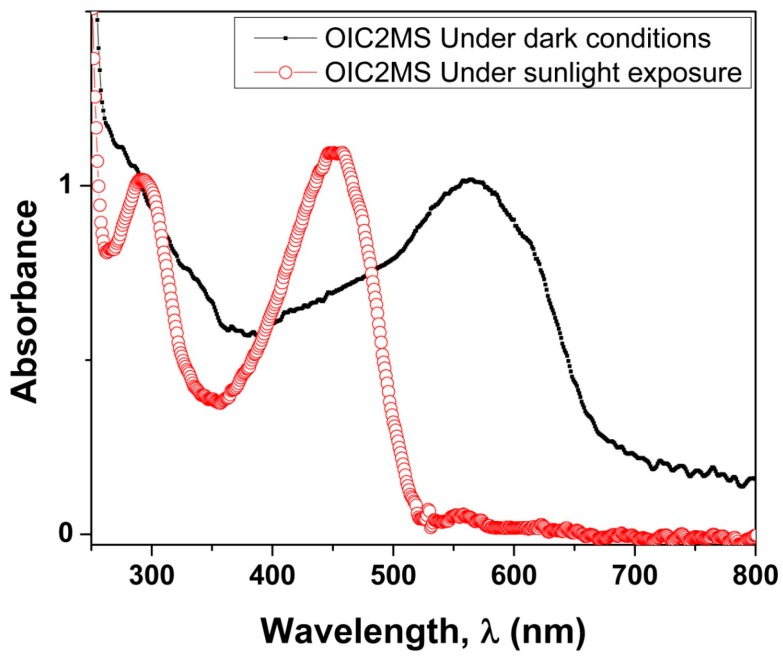
Absorption spectrum of **OIC2MS** under dark conditions without photo irradiation (red-open line) and photochromic effect of **OIC2MS** in chloroform upon sunlight irradiation (black-solid line) at room temperature.

The chloroform solution of **OIC2MS** turned to pale violet from pale yellow upon sunlight exposure over a period of 2.0 min, and a new absorption band appeared around 570 nm as shown in Figure 6 (solid line); however, there is no such of photochromic change under dark conditions (Figure 6, red line).

When the solution was kept in the dark condition, the pale violet color changed again into its original color. This is probably generated by a reversible transformation between isomers that absorb at different wavelengths [3]. As in the case of the chemical characterization, similar optical properties were observed irrespective of the synthesis method.

### 2.3. DFT Theoretical Studies

**Figure 7:**
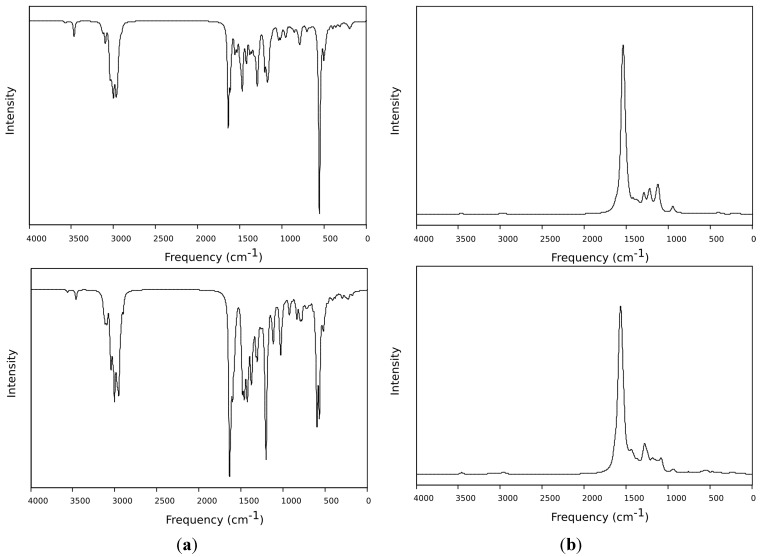
(**a**) IR spectra of **OIC1MS** (above) and **OIC2MS** (below) calculated at the PBEPBE/6-31G* level of theory. (**b**) Raman spectra of **OIC1MS** (above) and **OIC2MS** (below) calculated with the same methodology as the IR.

DFT was used at the PBEPBE/6-31G* level to simulate the infrared spectra, showing that there is a close resemblance with the experimental ones of both **OIC1MS** and **OIC2MS** molecules (Figure 7a). In particular, the imine bond signals are observed at 1630 cm^−1^ and 1645 cm^−1^ for **OIC1MS** and **OIC2MS** respectively, but the signal is mixed with contributions from the bending modes of the amine group. In the case of the Raman spectra, the signals arise mainly from stretching of the aromatic ring bonds with some contributions from stretching of the imine bonds (Figure 7b).

**Figure 8:**
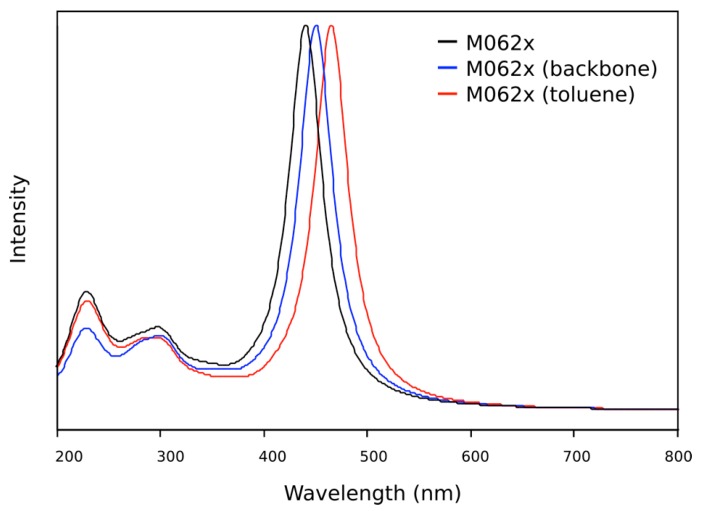
Theoretical UV/visible spectra of the OIC1MS molecule calculated with the M062x functional. The calculation was performed with the whole molecule without solvent (black line), with the aromatic backbone without solvent (blue line) and with the aromatic backbone and the implicit solvent parameters for toluene (red line).

UV/visible spectra were also calculated for **OIC1MS** and **OIC2MS** using the TD-DFT method. The PCM method was employed to see the effect of different solvents; however, it was found that agreement between the calculated and experimental spectra is not improved (see Figure 8). Moreover, the substitution of the aliphatic chains by methyl groups (leaving only the backbone) does not significantly change the UV/visible spectrum (see Figure 8). This is expected as the spectra of these compounds arise from electronic transitions in the aromatic backbone.

**Figure 9:**
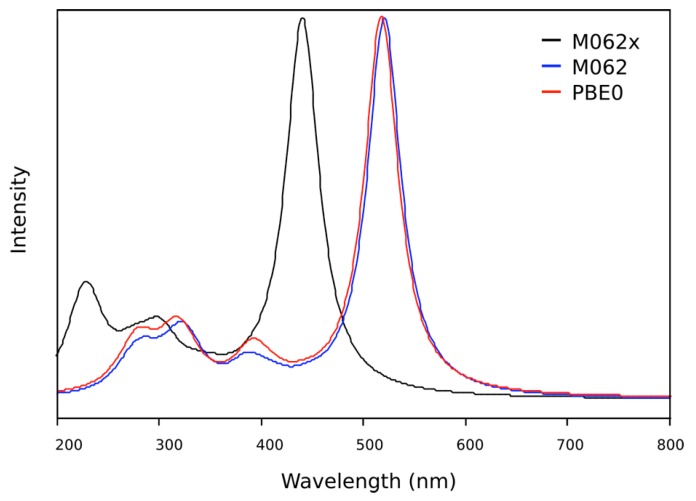
Gaseous state TD-DFT UV/visible spectra of **OIC1MS** calculated using the M062x functional (black line), M06 functional (blue line) and PBE0 functional (red line).

The TD-DFT calculations reproduce well the experimental spectrum for the OIC1MS molecule. The best agreement is obtained for the molecule in the gas phase with the M062x functional. Other functionals does not give consistent results (Figure 9) in agreement with reported studies of these functionals [11]. The main peak is located around 440 nm which is quite close to the experimental value (Figure 4a). A list of the main electronic excitations is given in Table 2.

**Table 2:** Electronic excitations of **OIC1MS** with oscillator strengths (>0.1) and major contributions (above 10%).

Wavelength (nm)	Oscillator Strength	Major Contributions
435.6	3.1	H-1→L+1 (13%), HOMO→LUMO (73%)
348.7	0.1	H-2→LUMO (42%), H-1→L+1 (19%)
298.5	0.3	H-7→LUMO (16%)
275.8	0.2	H-2→LUMO (30%)
221.1	0.2	H-6→L+1 (16%), H-2→L+2 (22%)
218.3	0.1	H-6→L+1 (52%), H-2→L+2 (11%)
215.2	0.1	H-1→L+7 (25%), HOMO→L+7 (18%), HOMO→L+8 (14%)
212.9	0.1	H-3→L+2 (20%), HOMO→L+7 (16%)

**Figure 10:**
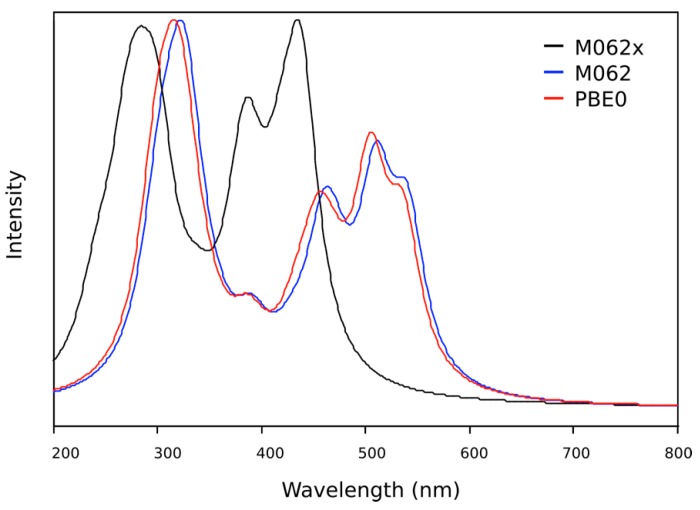
TD-DFT UV/visible spectrum of **OIC2MS**, calculated for the gaseous state using the M062x functional (black line), M06 functional (blue line) and PBE0 functional (red line).

For the **OIC2MS** molecule, only the backbone was considered in the TD-DFT calculations. The M062x functional performed considerably better than M06 and PBE0 (Figure 10). Unlike the **OIC1MS** molecule, **OIC2MS** shows two absorption bands (286 and 438 nm) in the theoretical spectrum. This qualitatively agrees with the experimental spectra (Figure 4b), and the main electronic excitations are given in Table 3.

**Figure 11:**
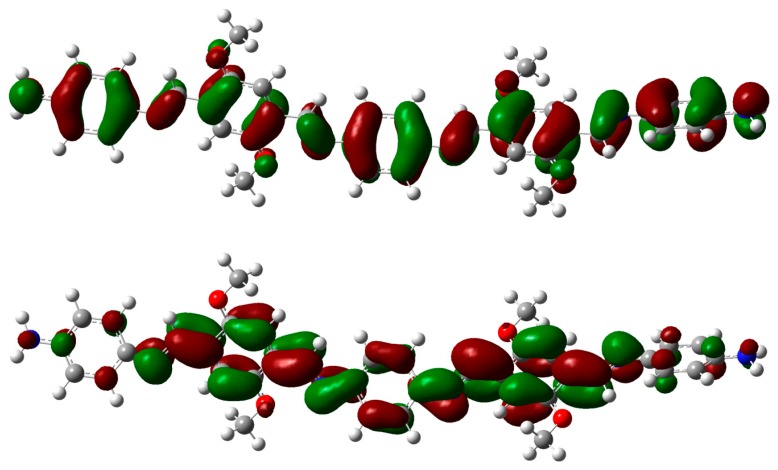
Molecular orbitals: HOMO (top) and LUMO (bottom) of **OIC1MS** calculated at the M062X/6-31G* level of theory. The aliphatic chains were removed, and the geometries were optimized as described in the text.

The main electronic transitions for both molecules were analyzed using the localization of molecular orbitals on the structure, calculated at the M062X/6-31G* level. In the calculated UV/visible spectrum of **OIC1MS**, the prominent peak appears around 440 nm, identified with the HOMO→LUMO (π→π*) electronic transition, whose contribution to the first excited state is around 73% (see Table 2). HOMO and LUMO orbitals (Figure 11) show that the electron density is partially transferred from the HOMO (localized over the whole aromatic backbone) to the three central phenyl rings.

**Table 3:** Electronic excitations for **OIC2MS** with oscillator strengths (>0.1) and major contributions (above 10%).

Wavelength (nm)	Oscillator Strength	Major Contributions
426.4	1.13	H-2→LUMO (13%), H-1→L+1 (13%), HOMO→LUMO (44%)
409.2	0.43	H-2→LUMO (17%), H-1→LUMO (23%), HOMO→L+1 (26%)
380.2	0.47	H-2→LUMO (20%), H-1→L+1 (22%)
371.0	0.53	H-2→LUMO (16%), H-2→L+1 (40%)
329.7	0.13	H-4→LUMO (38%), H-3→L+1 (23%)
300.0	0.23	H-14→L+1 (10%), H-1→LUMO (14%)
295.1	0.23	H-17→LUMO (11%), H-15→LUMO (10%), H-1→LUMO (11%), H-1→L+1 (10%), HOMO→L+1 (12%)
291.1	0.18	H-2→LUMO (11%), H-1→LUMO (13%), H-1→L+1 (12%), HOMO→LUMO (18%)
283.1	0.10	H-1→L+1 (20%), HOMO→L+1 (25%)
281.6	0.26	H-1→L+6 (10%), HOMO→L+6 (15%)
276.2	0.23	H-1→L+6 (17%), HOMO→L+6 (14%)
266.7	0.22	H-1→L+3 (10%), HOMO→L+2 (15%), HOMO→L+3 (10%)
261.7	0.28	H-2→L+2 (25%)

**Figure 12:**
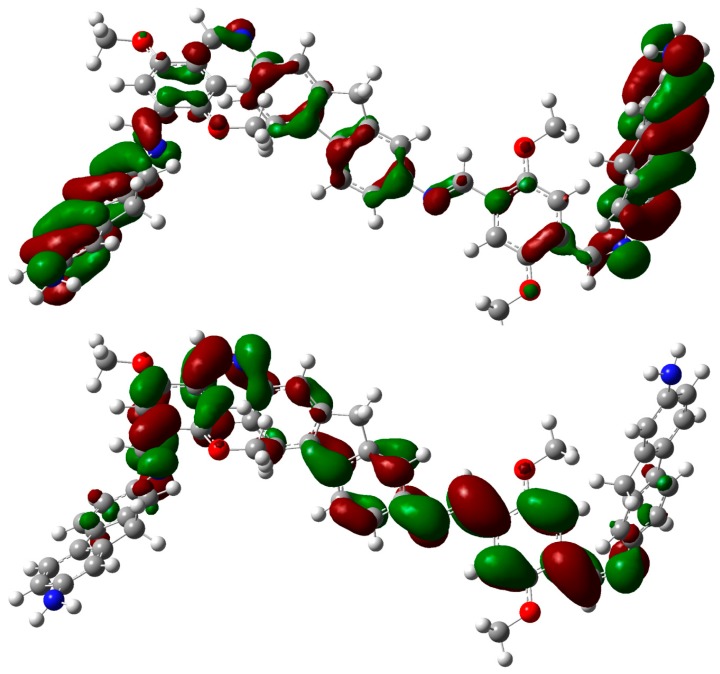
Molecular orbitals: HOMO (top) and LUMO (bottom) for **OIC2MS** calculated at the M062X/6-31G* level of theory. The aliphatic chains were removed and the geometries were optimized as described in the text.

For the **OIC2MS** molecule, an intense band was observed around 438 nm, identified as the HOMO→LUMO (π→π*) transition, whose contribution to the first excited state is around 44%. This indicates that in addition to the HOMO→LUMO transition, there are other relevant transitions contributing to the band (see Table 3). Figure 12 shows that electron density from the HOMO is localized over the two outer fluorene groups, and it is partially transferred to the two inner phenyl rings and the central fluorene group in the first excited state. The small overlap between the HOMO and LUMO orbitals shows that the charge transfer is more pronounced for **OIC2MS** compared to that for the **OIC1MS** molecule.

## 3. Experimental

### 3.1. General

Solvents and reagents were purchased as reagent grade and used without further purification. Toluene was distilled over calcium chloride. ^1^H- and ^13^C-NMR were recorded on a Varian 400 MHz NMR spectrometer with tetramethylsilane (TMS) as an internal reference. Infrared (IR) spectra were measured on a Nicolet FT- Magna 550 spectrophotometer. Elemental analysis was determined on a Perkin Elmer 2400 unit. UV spectra were taken on a Perkin Elmer Lambda XLS. Mass spectral fragments were determined on Agilent Triple Quad 6410 LC-MS.

### 3.2. Theoretical Calculations

Simulated annealing of the **OIC1MS** and **OIC2MS** molecules was carried out with the DFTB+ software [12]. In this step five random conformers of each molecule were heated from 300 K to 900 K and cooled again to 300 K using a linear scheduling. The most stable conformer of each molecule was locally optimized and characterized through a normal mode analysis in order to determine maximum or minimum in the potential energy surface. The infrared and Raman spectra were calculated within the harmonic approximation applying a convolution with Lorentzian functions to simulate the experimental spectra. These calculations were performed with DFT theory at the PBEPBE/6-31G* level. The UV/visible spectra were calculated for **OIC1MS** and **OIC2MS** with TD-DFT theory at the PBE0/cc-pVTZ, M06/cc-pVTZ and M062X/cc-pVTZ levels. The calculated spectrum was obtained through convolution of the first 30 electronic excitations with Lorentzian functions. All DFT and TD-DFT calculations were performed with the Gaussian 09 [13] software.

### 3.3. Mechanosynthesis

#### Experimental Procedure 

Mechanosynthesis was carried out in a Spex 8000D mixer mill in D2 tool steel vials with hardened steel balls. 2,5-bis(octyloxy)terephtaldehyde (0.1 g, 0.256 mmol) and 1,4-phenylendiamine (0.068 g, 0.640 mmol) or 2,5-diaminofluorene (0.125 g, 0.640 mmol) were added to the vial, and they were milled for 90 min. Then the vial was left to reach room temperature and opened. The reaction product was dissolved in CHCl_3_, washed three times with water, and the organic phase was dried with Na_2_SO_4_. The crude product was purified by precipitation using hexane.

*4,4'-(((1E,1'E)-(((1E,1'E)-(1,4-phenylenebis(azanylylidene))bis(methanylylidene))bis(2,5-bis(octyloxy)-1,4-phenylene))bis(methanylylidene))bis(azanylylidene))dianiline* (**OIC1MS**): Yield 90%; orange powder; Td 152 °C; Elemental Analysis: C_66_H_92_N_6_O_4_; Calculated % C = 76.70, % H = 8.97, % N = 8.13. Observed. % C = 76.65, % H = 8.92, % N = 7.85. FT-IR/ATR ν (cm-1): 3316, 3204, 3028, 2926, 2855; 1610, 1583, 1509; Raman (cm^−1^): 1555, 1578; ^1^H-NMR (CDCl_3_) ppm: 8.9 (b, 4H, HC=N), 7.82–6.50 (m, 16H, Ar), 4.20 (t, 8H, *J* = 6.7 Hz, CH_2_α-O), 3.71 (b, 4H, NH_2_), 1.92–1.20 (b, 48H, -CH_2_), 0.89 (b, 12H, CH_3_); ^13^C-NMR (CDCl_3_) ppm: 152.15–151.52 (C=N), 144.12 (Ar), 142.51 (Ar), 127.21 (Ar), 121.59(Ar), 121.05 (Ar), 115.68 (Ar), 114.53 (Ar), 109.16 (Ar), 68.13 (CH_2_α-O), 30.77 (-CH_2_-), 28.24 (-CH_2_-), 25.13 (-CH_2_-), 21.65 (-CH_2_-), 13.10 (–CH_3_); MS (*m*/*z*): 1033 [M^+^].

*7,7'-(((1E,1'E)-(((1E,1'E)-((9H-fluorene-2,7-diyl)bis(azanylylidene))bis(methanylylidene))bis(2,5-bis-(octyloxy)-1,4-phenylene))bis(methanylylidene))bis(azanylylidene))bis(9H-fluoren-2-amine)* (**OIC2MS**): Yield 89%; orange powder; Td 242 °C; Elemental Analysis: C_87_H_104_N_6_O_4_; Calculated % C = 80.52, % H = 8.08, % N = 6.48. Experimental. % C = 79.85, % H = 7.99, % N = 6.44. FT-IR/ATR ν (cm^−1^): 3465, 3329, 3007, 2926, 2854, 1615, 1589, 1491; Raman [cm^−1^]: 1556, 1584; ^1^H-NMR (CDCl_3_) ppm: 9.03 (b, 4H, HC=N), 8.0–6.50 (m, 22 H, Ar), 4.16–3.54 (m, 4H, NH_2_), (8H, CH_2_α-O), (6H, cyclopentadiene), 1.97–1.17 (b, 8H, CH_2_), 1.63–1.44 (m, 48H, CH_2_), 0.87 (b, 12H, CH_3_); ^13^C-NMR (CDCl_3_) ppm: 152.15–151.52 (C=N),144.12 (Ar), 142.51 (Ar), 127.21 (Ar), 121.59 (Ar), 121.05 (Ar), 115.68 (Ar), 114.53 (Ar), 109.16 (Ar), 68.13 (CH_2_α-O), 30.77 (-CH_2_-), 28.24 (-CH_2_-), 25.13 (-CH_2_-), 21.65 (-CH_2_-), 13.10 (–CH_3_); MS (*m*/*z*): [M-726], [M-923], [M-1100].

### 3.4. Conventional Synthesis

#### Experimental procedure 

In a 100 mL bottom flask, 2,5-bis(octyloxy)terephtaldehyde (0.1 g, 0.256 mmol) and 1,4-phenylendiamine (0.068 g, 0.640 mmol) or 2,5-diaminofluorene (0.125 g, 0.64 mmol) were dissolved in toluene (20 mL). The reaction mixture was kept under an argon atmosphere with magnetic stirring, and heated at reflux for 24 h. The crude product was purified by precipitation using hexane.

*4,4'-(((1E,1'E)-(((1E,1'E)-(1,4-phenylenebis(azanylylidene))bis(methanylylidene))bis(2,5-bis(octyl-oxy)-1,4-phenylene))bis(methanylylidene))bis(azanylylidene))dianiline* (**OIC1**): Yield 87%; orange powder; Td 152 °C; Elemental Analysis: C_66_H_92_N_6_O_4_; Calculated % C = 76.70, % H = 8.97, % N = 8.13. Observed. % C = 76.68, % H = 8.94, % N = 8.0. FT-IR/ATR ν (cm^−1^): 3316, 3204, 3028, 2926, 2855; 1610, 1583, 1509; Raman (cm^−1^): 1555, 1578; ^1^H-NMR (CDCl_3_) ppm: 8.9 (b, 4H, HC=N), 7.82–6.50 (m, 16H, Ar), 4.20 (t, 8H, *J* = 6.7 Hz, CH_2_α-O), 3.71 (b, 4H, NH_2_), 1.92–1.20 (b, 48H, -CH_2_), 0.89 (b, 12H, CH_3_); ^13^C-NMR (CDCl_3_) ppm: 152.15–151.52 (C=N), 144.12 (Ar), 142.51 (Ar), 127.21 (Ar), 121.59 (Ar), 121.05 (Ar), 115.68 (Ar), 114.53 (Ar), 109.16 (Ar), 68.13 (CH_2_α-O), 30.77 (-CH_2_-), 28.24 (-CH_2_-), 25.13 (-CH_2_-), 21.65 (-CH_2_-), 13.10 (–CH_3_); MS (*m*/*z*): 1033 [M+].

*7,7'-(((1E,1'E)-(((1E,1'E)-((9H-fluorene-2,7-diyl)bis(azanylylidene))bis(methanylylidene))bis(2,5-bis(octyloxy)-1,4-phenylene))bis(methanylylidene))bis(azanylylidene))bis(9H-fluoren-2-amine)* (**OIC2**): Yield 81%; orange powder; Td 242 °C; FT-IR/ATR ν (cm^−1^): 3465, 3329, 3007, 2926, 2854, 1615, 1589, 1491; Raman [cm^−1^]: 1556, 1584; ^1^H-NMR (CDCl_3_) ppm: 9.03 (b, 4H, HC=N), 8.0–6.50 (m, 22 H, Ar), 4.16–3.54 (m, 4H, NH_2_), (8H, CH_2_α-O), (6H, cyclopentadiene), 1.97–1.17 (b, 8H, CH_2_), 1.63–1.44 (m, 48H, CH_2_), 0.87 (b, 12H, CH_3_); ^13^C-NMR (CDCl_3_) ppm: 152.15–151.52 (C=N), 144.12 (Ar), 142.51 (Ar), 127.21 (Ar), 121.59 (Ar), 121.05 (Ar), 115.68 (Ar), 114.53 (Ar), 109.16 (Ar), 68.13 (CH_2_α-O), 30.77 (-CH_2_-), 28.24 (-CH_2_-), 25.13 (-CH_2_-), 21.65 (-CH_2_-), 13.10 (–CH_3_); MS (*m*/*z*): [M-726], [M-923], [M-1100].

## 4. Conclusions

In the present work, mechanosynthesis is shown to be an efficient method for the synthesis of the phenyleneimine pentamers as it requires less time than the conventional method, and no solvent or inert atmosphere is used for the synthesis. The product **OIC2MS** exhibits photochromic properties compared to **OIC1MS**. This is probably caused by reversible transformations between isomers that absorb at different wavelengths. These results were confirmed with DFT theoretical simulations.

## Supplementary Materials

Supplementary materials can be accessed at: http://www.mdpi.com/1420-3049/20/04/5440/s1.
