# Supplementary material for: Synthesis of Photochromic Oligophenylenimines: Optical and Computational Studies
Source: Molecules. 2015 Mar 27;20(4):5440–55. doi: 10.3390/molecules20045440 (PMC6272491; doi:10.3390/molecules20045440)

Supplementary File

Fragment Patron OIC1C and OIC1MS

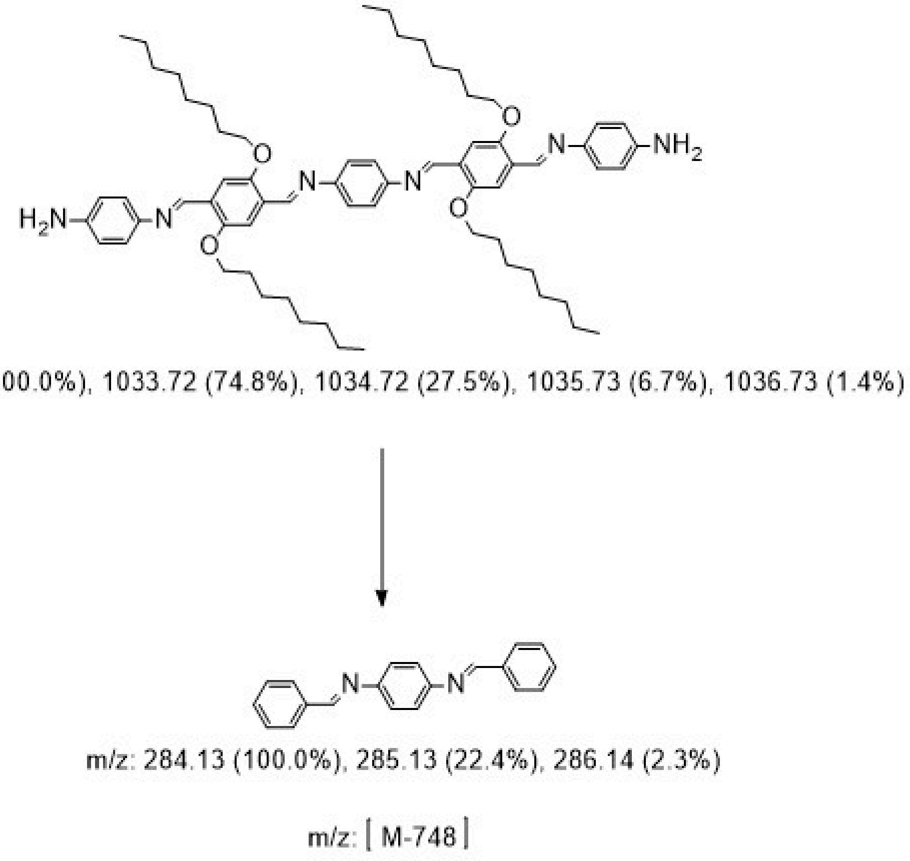

Fragment patron OIC1C and OIC1MS

Fragment Patron OIC2C and OIC2MS

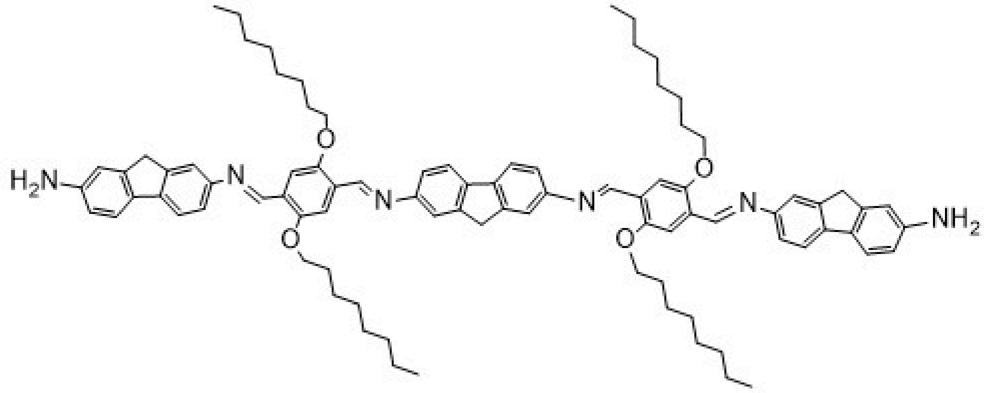

m/z: 1296.81 (100.0%), 1297.82 (95.4%), 1298.82 (45.9%), 1299.82 (15.8%), 1300.83 (3.2%), 1297.81 (2.2%), 1298.81 (2.1%)

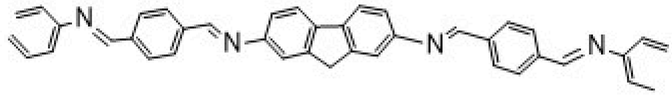

m/z: 570.28 (100.0%), 571.28 (45.1%), 572.29 (9.3%), 573.29 (1.3%)

[M-726]

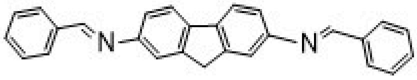

m/z: 372.16 (100.0%), 373.17 (29.4%), 374.17 (4.2%)

[M-923]

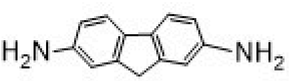

m/z: 196.10 (100.0%), 197.10 (14.8%)

[M-100]

Qualitative Analysis Report of OIC1C

Qualitative Analysis Report

**Data Filename**	OT292_ID_1_CHCL3.d	**Sample Name**	OIC1C
**Sample Type**	Sample	**Position**	P1-F4
**Instrument Name**	Instrument 1	**User Name**	
**Acq Method**	ESI_POS_SCAN_100ACN-ID.m	**IRM Calibration Status**	Success
**DA Method**	ESI-NEG_FLAV_MGV.m	**Comment**	

User Chromatograms

Fragmentor Voltage 135 **Collision Energy** 0 **Ionization Mode** Esi

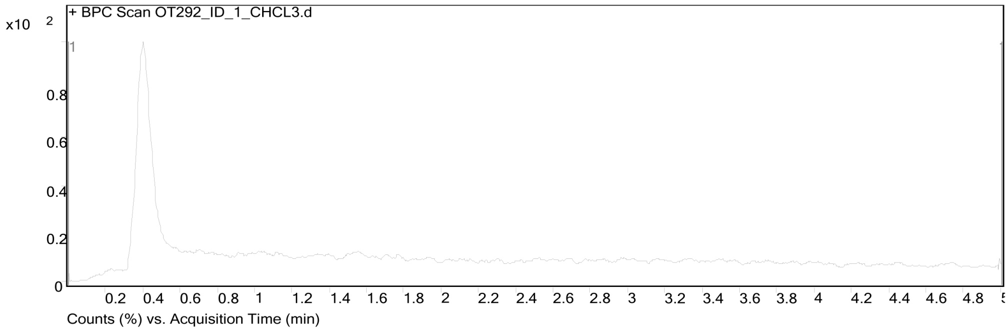

User Spectra

**Fragmentor Voltage**	**Collision Energy**	**Ionization Mode**
135	0	Esi

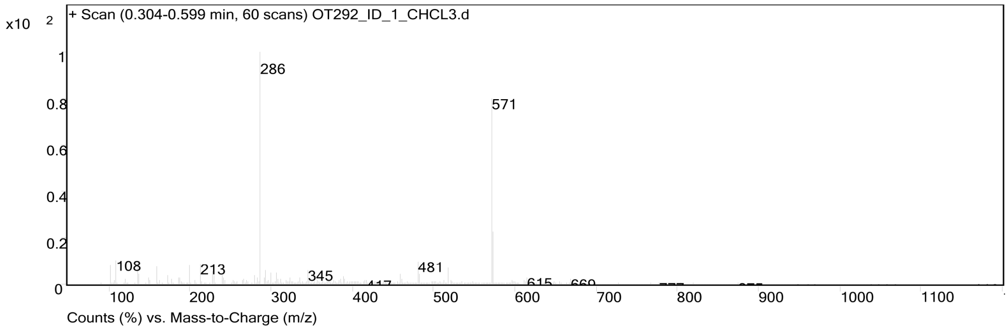

Peak List

m/z	z	Abund.
102		22194
108		28342
109		26047
135	2	21836
213		24040
286		272724
481		26897
571		218979
572	1	25956
572	1	62412

OIC1MS-1H-NMR

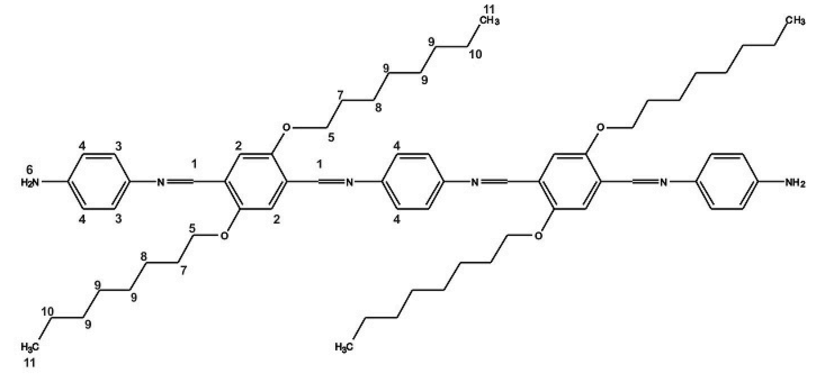

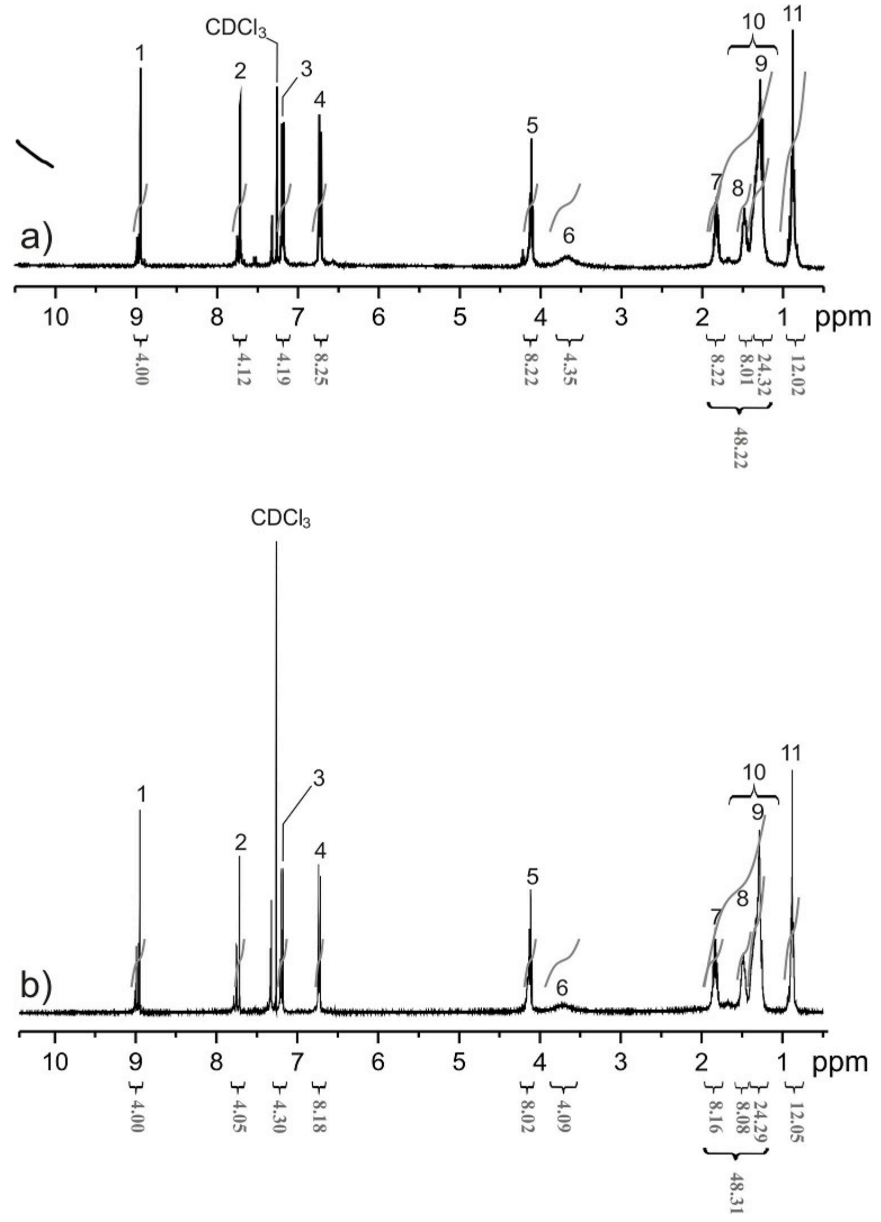

Qualitative Analysis Report of OIC1MS

Qualitative Analysis Report

**Data Filename**	OT290_ID_CHCL3_1.d	**Sample Name**	OIC1MS
**Sample Type**	Sample	**Position**	P1-F2
**Instrument Name**	Instrument 1	**User Name**	
**Acq Method**	ESI_POS_SCAN_100ACN-ID.m	**IRM Calibration Status**	Success
**DA Method**	ESI-NEG_FLAV_MGV.m	**Comment**	

User Chromatograms

Fragmentor Voltage 135 **Collision Energy** 0 **Ionization Mode** Esi

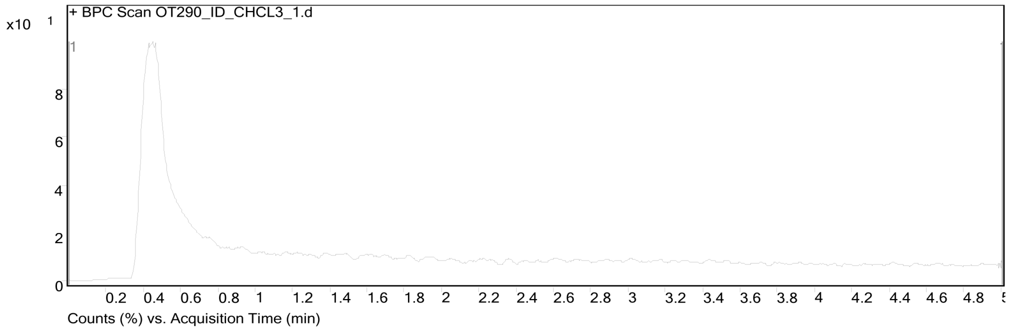

User Spectra

**Fragmentor Voltage**	**Collision Energy**	**Ionization Mode**
135	0	Esi

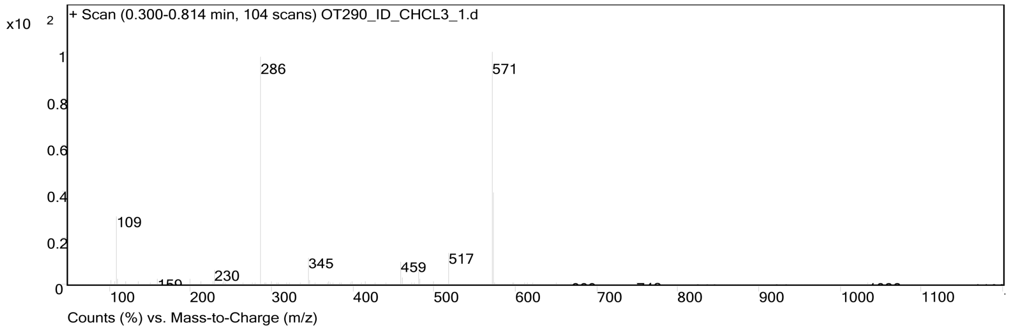

Peak List

m/z	z	Abund.
108		105085
109	1	268831
230		52775
286		895358
345		104480
459	1	89500
517		125566
571		915530
572	1	363141
573	1	68925

Qualitative Analysis Report of OIC2C

Qualitative Analysis Report

**Data Filename**	OT294_ID_CHCL3_1.d	**Sample Name**	OIC2C
**Sample Type**	Sample	**Position**	P1-F6
**Instrument Name**	Instrument 1	**User Name**	
**Acq Method**	ESI_POS_SCAN_100ACN-ID.m	**IRM Calibration Status**	Success
**DA Method**	ESI-NEG_FLAV_MGV.m	**Comment**	

User Chromatograms

Fragmentor Voltage 135 **Collision Energy** 0 **Ionization Mode** Esi

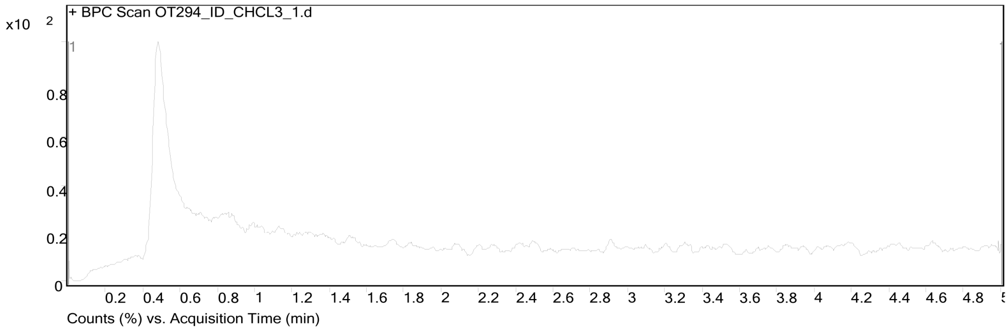

User Spectra

**Fragmentor Voltage**	**Collision Energy**	**Ionization Mode**
135	0	Esi

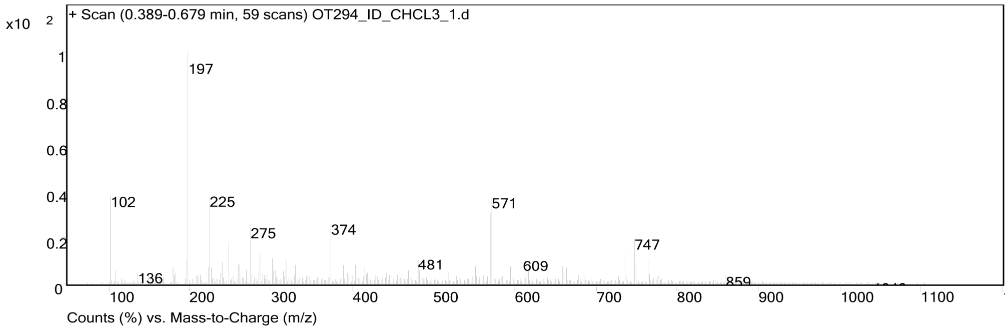

Peak List

m/z	z	Abund.
102		35402
197	1	93105
225	1	35414
247		16949
275	1	22885
374		24116
569		28743
571		19448
571	1	34728
747		18376

Qualitative Analysis Report of OIC2MS

Qualitative Analysis Report

**Data Filename**	OT293_ID_1_CHCL3.d	**Sample Name**	0IC2MS
**Sample Type**	Sample	**Position**	P1-F5
**Instrument Name**	Instrument 1	**User Name**	
**Acq Method**	ESI_POS_SCAN_100ACN-ID.m	**IRM Calibration Status**	Success
**DA Method**	ESI-NEG_FLAV_MGV.m	**Comment**	

User Chromatograms

Fragmentor Voltage 135 **Collision Energy** 0 **Ionization Mode** Esi

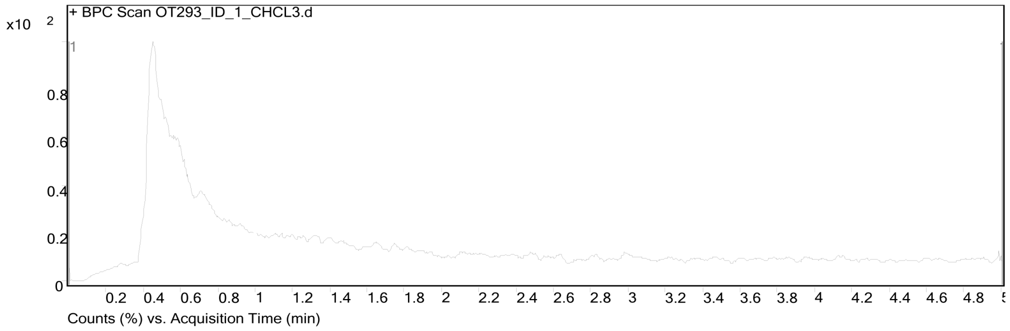

User Spectra

Fragmentor Voltage	Collision Energy	Ionization Mode
135	0	Esi

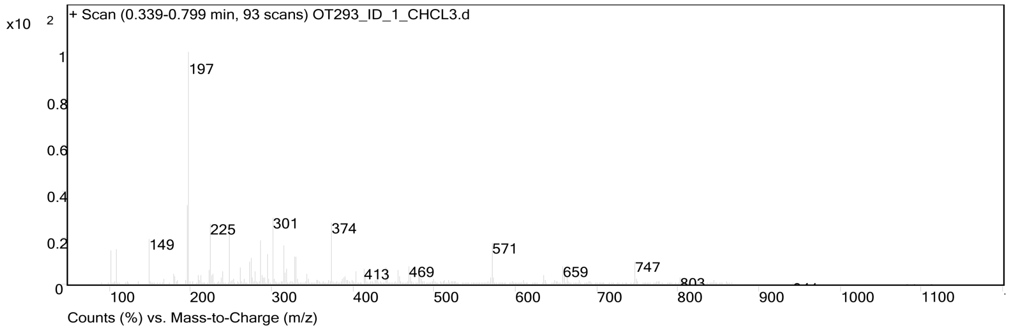

Peak List

m/z	z	Abund.
149		29365
196		51949
197	2	152117
225	2	39366
247	1	36498
287		29097
301	1	43953
315	1	25483
374		40382
571		27394

OIC2MS-1H-NMR

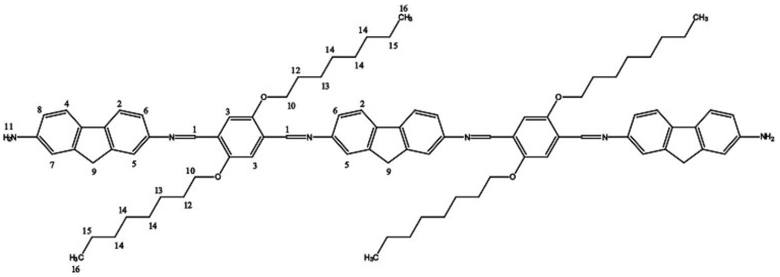

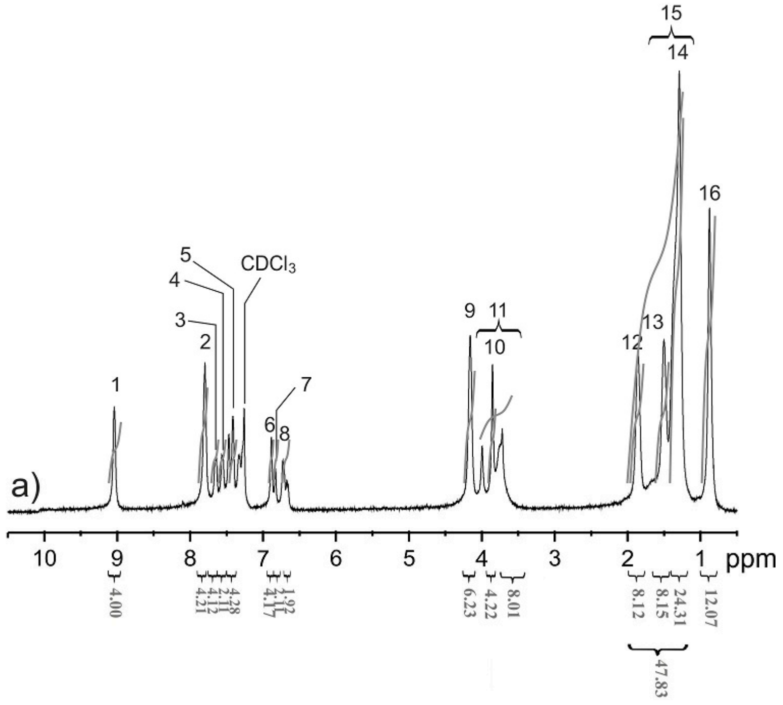

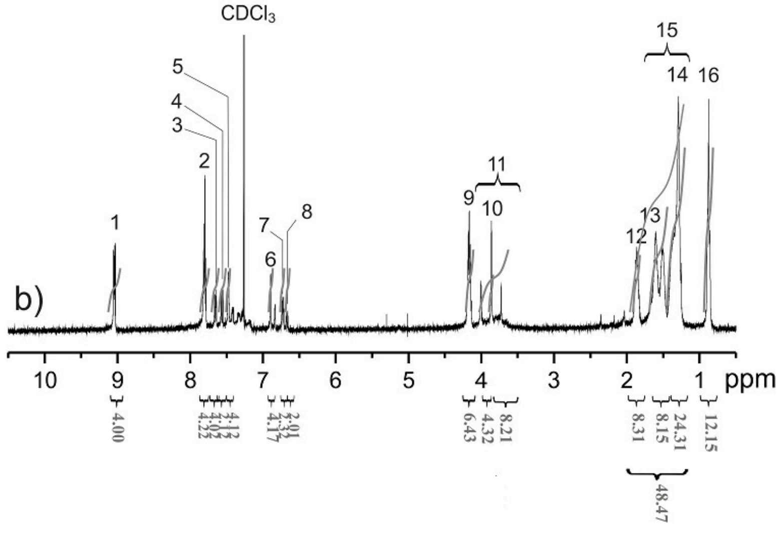

Supplement: Supplementary file 1 [file molecules-20-05440-s001.pdf]
